# Outcomes and Safety of History-Based Screening for Medication Abortion: A Retrospective Multicenter Cohort Study

**DOI:** 10.1001/jamainternmed.2022.0217

**Published:** 2022-03-21

**Authors:** Ushma D. Upadhyay, Elizabeth G. Raymond, Leah R. Koenig, Leah Coplon, Marji Gold, Bliss Kaneshiro, Christy M. Boraas, Beverly Winikoff

**Affiliations:** 1 Department of Obstetrics, Gynecology, & Reproductive Sciences, University of California, San Francisco; 2 Gynuity Health Projects, New York, New York; 3 Department of Epidemiology and Biostatistics, University of California, San Francisco; 4 Maine Family Planning, Augusta; 5 RHEDI/Montefiore Medical Center, Bronx, New York; 6 University of Hawaii, Honolulu, Hawaii; 7 Planned Parenthood North Central States, Minneapolis, Minnesota

## Abstract

**Importance:**

Screening for medication abortion eligibility typically includes ultrasonography or pelvic examination. To reduce physical contact during the COVID-19 pandemic, many clinicians stopped requiring tests before medication abortion and instead screened patients for pregnancy duration and ectopic pregnancy risk by history alone. However, few US-based studies have been conducted on the outcomes and safety of this novel model of care.

**Objective:**

To evaluate the outcomes and safety of a history-based screening, no-test approach to medication abortion care.

**Design, Setting, and Participants:**

This retrospective cohort study included patients obtaining a medication abortion without preabortion ultrasonography or pelvic examination between February 1, 2020, and January 31, 2021, at 14 independent, Planned Parenthood, academic-affiliated, and online-only clinics throughout the US.

**Exposures:**

Medications for abortion provided without preabortion ultrasonography or pelvic examination and dispensed to patients in person or by mail.

**Main Outcomes and Measures:**

Effectiveness, defined as complete abortion after 200 μg of mifepristone and up to 1600 μg of misoprostol without additional intervention, and major abortion-related adverse events, defined as hospital admission, major surgery, or blood transfusion.

**Results:**

The study included data on 3779 patients with eligible abortions. The study participants were racially and ethnically diverse and included 870 (23.0%) Black patients, 533 (14.1%) Latinx/Hispanic patients, 1623 (42.9%) White patients, and 327 (8.7%) who identified as multiracial or with other racial or ethnic groups. For most (2626 [69.5%]), it was their first medication abortion. Patients lived in 34 states, and 2785 (73.7%) lived in urban areas. In 2511 (66.4%) abortions, the medications were dispensed in person; in the other 1268 (33.6%), they were mailed to the patient. Follow-up data were obtained for 2825 abortions (74.8%), and multiple imputation was used to account for missing data. Across the sample, 12 abortions (0.54%; 95% CI, 0.18%-0.90%) were followed by major abortion-related adverse events, and 4 patients (0.22%; 95% CI, 0.00%-0.45%) were treated for ectopic pregnancies. Follow-up identified 9 (0.40%; 95% CI, 0.00%-0.84%) patients who had pregnancy durations of greater than 70 days on the date the mifepristone was dispensed that were not identified at screening. The adjusted effectiveness rate was 94.8% (95% CI, 93.6%-95.9%). Effectiveness was similar when medications were dispensed in person (95.4%; 95% CI, 94.1%-96.7%) or mailed (93.3%; 95% CI, 90.7%-95.9%).

**Conclusions and Relevance:**

In this cohort study, screening for medication abortion eligibility by history alone was effective and safe with either in-person dispensing or mailing of medications, resulting in outcomes similar to published rates of models involving ultrasonography or pelvic examination. This approach may facilitate more equitable access to this essential service by increasing the types of clinicians and locations offering abortion care.

## Key Points

**Question:**

What are the outcomes and safety of medication abortion care provided to patients screened for eligibility by history alone without ultrasonography or pelvic examination?

**Findings:**

In this multicenter cohort study of 3779 patients with eligible abortions, 95% of abortions were complete without additional medical intervention, and 0.54% were followed by a major abortion-related adverse event.

**Meaning:**

This study provides evidence that screening patients for medication abortion using history alone maintains high effectiveness and low risk; this screening approach may facilitate more equitable access to abortion care by enabling a wider variety of clinicians to offer this essential service.

## Teaser

This cohort study evaluates the outcomes and safety of a history-based screening, no-test approach to medication abortion care.

## Introduction

Each year nearly 1 million people in the US seek an induced abortion.^1^ Medication abortion with mifepristone and misoprostol is currently approved by the US Food and Drug Administration (FDA) for use through 70 days of pregnancy,^2^ although based on evidence, many clinicians offer it up to 77 days.^3,4^ Typically, clinicians perform ultrasonography or a pelvic examination before treatment to determine the duration and location of the pregnancy. However, during the COVID-19 pandemic, some clinics relied on patient history alone, without ultrasonography or pelvic examination, to reduce physical contact.^5,6,7,8^ In 2020, the FDA temporarily relaxed its in-person dispensing requirement on mifepristone for the duration of the pandemic.^9^ By October 2020, guidelines issued by the Society of Family Planning,^10^ the American College of Obstetricians and Gynecologists^10^ and the National Abortion Federation^11^ had been amended to endorse this no-test approach, providing abortion medications without ultrasonography or other facility-based tests.^12^ Together, these changes enabled the emergence of several new online services that offered medication abortion entirely remotely using telemedicine and mail.^13,14^

Several studies, primarily in the UK and Canada, have concluded that history-based screening for medication abortion is safe and effective.^14,15,16,17,18^ The largest, which included 18 435 medication abortions in the UK provided without screening ultrasonography, reported that 99% were complete without intervention, and serious adverse events occurred in 0.02%.^15^ An analysis of data from 425 participants in the TelAbortion study,^19^ a prospective multicenter study of medication abortions provided by telemedicine and mail conducted in the US, also found high effectiveness and safety in the subset screened by history alone, although these patients had lower effectiveness rates (94%) than those who had had pretreatment tests (98%). Another study among 141 patients of a new online service in California found an effectiveness rate of 95% and no major adverse events.^14^

Many people throughout the US face insurmountable financial, transportation, legal, and other barriers to reaching an abortion facility,^20^ and access to abortion care is geographically inequitable.^21,22^ Longer travel distance means increased logistical and emotional burdens and costs for gas or public transit fare, hotel stays, and loss of wages from time off work, as well as arranging for childcare. Access to patient-centered abortion care could be greatly improved if more primary care and other clinicians could prescribe abortion medications locally to their patients without specialized equipment and if abortion medications could be dispensed by brick-and-mortar and mail-order pharmacies.

We conducted a multicenter retrospective cohort study assessing the effectiveness and safety of using history-based screening alone for medication abortion care among a large sample of US patients from diverse clinics. This article summarizes the protocols that the clinics adopted, describes the characteristics of patients who had no-test abortions, and estimates the effectiveness and safety of no-test medication abortion. We also compare the effectiveness of abortions with medications dispensed in-person to those dispensed by mail to patients.

## Methods

Between May 2020 and January 2021, we recruited US clinics through webinars, professional email distribution lists, and personal contacts, inviting any facility that offered medication abortion using history-based screening for at least some patients to join the study. We included clinics that offered history-based screening through the TelAbortion study (NCT02513043 and NCT04599725).^23,24^ Each clinic completed a survey on its medication abortion protocol, including eligibility criteria and screening procedures. We followed the Strengthening the Reporting of Observational Studies in Epidemiology (STROBE) reporting guidelines for cohort studies. Allendale Institutional Review Board and University of California, San Francisco Institutional Review Board provided ethical approvals. Because this study was a retrospective medical record review, and thus presented no more than minimal risk, the institutional review boards waived the need for patient informed consent.

Staff at participating clinics abstracted medical records of all medication abortions in which both mifepristone and misoprostol were dispensed without pretreatment ultrasonography or pelvic examination during a defined subperiod between February 1, 2020, and January 31, 2021. Clinic staff were required to be familiar with their clinic’s protocols and received a 1-hour training with 1 or 2 of the study coauthors (L.R.K. and sometimes E.G.R.). Staff entered data into a secure online REDCap database,^25,26^ including patient age, race and ethnicity, abortion payment type, zip codes, details about mifepristone and misoprostol provision, and events after provision, such as timing of mifepristone and misoprostol administration, unscheduled clinical contacts, treatments, diagnosis and treatment of ectopic pregnancy, last known status of pregnancy (viable, nonviable, or unknown), and confirmatory test results. Race and ethnicity categories included Black, Latinx/Hispanic, White, multiracial and other, and unknown. The “other” category included Alaska Native, American Indian, Asian, Asian Indian, Filipino, Guyanian, Guamanian/Chamorro, Japanese, Middle Eastern, Native American, Native Hawaiian, Pacific Islander, and Turkish. We used 2010 Rural-Urban Commuting Area (RUCA) codes^27^ to categorize participant zip codes into urban, suburban, and rural places.

We asked site staff to report the final outcome as determined by a clinician and as recorded in the medical record. Although we collected data on test results that supported the diagnosis of complete medication abortion, we did not systematically collect information on the criteria clinicians used to diagnose abortions as complete by history. The first 3 authors (U.D.U., E.G.R., and L.R.K.) reviewed selected cases to clean data and flagged any inconsistencies within a specific medical record. These records were then rereviewed by clinic staff, who made any needed corrections. Additionally, 4 clinician coauthors with experience in abortion provision (B.K., L.C., C.M.B., and M.G.) reviewed selected cases to resolve analysis outcomes.

### Outcomes

Effectiveness and safety were the primary outcomes of interest and were defined during a consensus meeting held in December 2020 with experts in medication abortion research and clinical care. We defined effectiveness as a binary measure of complete medication abortion after initial treatment without subsequent known intervention. Abortions that met any of the following 4 criteria were determined not to be complete: (1) the patient had an aspiration, dilation and evacuation, other procedure, or other surgical intervention; (2) the patient received greater than 200 mg of mifepristone, more than 1600 μg of misoprostol, or other uterotonic medications; (3) the patient received treatment for ectopic pregnancy; or (4) the patient had a viable pregnancy detected by ultrasonography at last contact and no known intervention.

Among abortions recorded as complete, we coded abortions as “complete by test” if the patient had a negative urine pregnancy test result, ultrasonography or pelvic examination showing no continuing pregnancy, an expected decline in serum beta human chorionic gonadotropin (β-hCG) level, a single posttreatment serum β-hCG value less than 500 mIU/mL at least 8 days after mifepristone dispensing,^28^ or clinician examination of intact fetus or fetal parts. We coded abortions as “complete by history” if the site staff indicated that the abortion was complete based on a symptom checklist or patient report, if at the time of a subsequent pregnancy the patient reported that the prior pregnancy had ended in abortion, or if the patient reported expulsion of intact fetus or fetal parts. After reviewing preliminary results, including a lower proportion of known outcomes and a lower effectiveness rate among the group who received the medications by mail rather than in person, we reviewed 230 records for which the abortion outcome was originally coded as unknown but included text pertaining to the abortion outcome in the notes field. The clinician authors reviewed all 230 of these records and recoded 67 of them as “complete by history” based on notes in the study database indicating that the treating clinician had no concern that the abortion was incomplete after phone, text, or email follow-up contact with the patient. The results presented herein incorporate the recoded data.

We defined safety as a binary measure of abortions not followed by a known abortion-related major adverse event, which was defined as hospital admission, blood transfusion, major surgery, including laparotomy and laparoscopy for ectopic pregnancy, or death. The clinician authors determined the relation of adverse events to the abortion by consensus.

We assessed 2 secondary outcomes: ectopic pregnancy and retrospective determination that the initial medication abortion treatment had been provided at greater than 70 days of gestation. While some clinics routinely offered medication abortion up to 77 days, we sought to identify any abortion where it was discovered only at follow-up that the initial medication was dispensed beyond the gestational limit on the FDA-approved mifepristone label.

### Statistical Analysis

We first described the characteristics of the clinics and their protocols. We then summarized patient and service delivery characteristics and compared the characteristics of patients for whom abortion outcomes were known and unknown.

We then used mixed-effects logistic regression models to produce effectiveness and safety rates using Stata’s margins and mimrgns commands. After finding no evidence of differential clustering by TelAbortion participation or by multiple abortions from the same patient, these models included only 1 random intercept to account for clinic-level clustering.

To estimate effectiveness outcomes, we originally conducted complete case analyses, examining unadjusted and adjusted effectiveness rates overall and by patient and service delivery characteristics among only those abortions with recorded outcomes. However, based on editor suggestion, we then conducted an analysis using multiple imputation by chained equations to account for missing outcomes due to loss to follow-up. We assumed data to be missing at random and imputed missing data for covariates and effectiveness and safety outcomes in the multivariable models using Stata’s mi impute command.^29^ Imputation models included effectiveness and safety outcome variables and age, race and ethnicity, residence, prior medication abortion, participation in the TelAbortion Study, method of mifepristone provision, and pregnancy duration. We computed 200 imputations to facilitate reproducibility for rare outcomes and combined the imputed results using Stata’s mi estimate command. We then used a multivariable mixed-effects logistic regression model adjusting for age, race and ethnicity, residence, prior medication abortion, participation in the TelAbortion Study, method of mifepristone provision, and pregnancy duration to estimate adjusted effectiveness rates. We also estimated rates of intervention and continuing pregnancy using multivariable mixed-effects logistic regression, adjusting for the same variables.

We performed 2 additional sensitivity analyses for the effectiveness outcomes. The first replicated the effectiveness analysis excluding patients whose outcomes were determined by history alone. The second sensitivity analysis was ad hoc, based on reviewer suggestion, among only those at 9 weeks or shorter gestation who initially received 800 μg of misoprostol. Both of these analyses were performed on the imputed data and adjusted for the same covariates as the primary efficacy analysis.

We then estimated the overall safety rate among all abortions with any follow-up information recorded, as well as the rates of each major adverse event type (blood transfusion, surgery, or hospital admission) without adjustment. Additionally, we estimated adjusted safety rates using the imputed data set, adjusting for the same covariates as in the effectiveness analysis. To facilitate model convergence for all rare outcomes (n < 9), the random intercept for clinic was omitted in the unadjusted analyses, and rates from the imputed data were calculated with random effects but without adjustment. We also described all ectopic pregnancies and the outcomes of cases found by posttreatment ultrasonography or examination of the fetus to have had pregnancy durations greater than 70 days at screening, but not identified at screening.

For both effectiveness and safety outcomes, we report both the unadjusted complete case analysis results and the adjusted multiple imputation analysis results. All rates reported in text are from the random-effects models calculated after imputation.

All analyses excluded patients who reported that they took neither mifepristone nor misoprostol. All analyses of patient data used the abortion, not patient, as the unit of analysis. All statistical tests were 2-tailed with significance set at .05. We used Stata, version 15.1 (StataCorp LLC).

## Results

### Participating Clinics

We received data from 15 clinics, representing independent, Planned Parenthood, academic-affiliated, and online-only clinics. We excluded 1 clinic that contributed only 3 abortions, leaving 14 clinics for analysis. Each clinic contributed a range of 11 to 981 eligible abortions; 5 provided no-test abortions both outside of and within the TelAbortion study, 1 provided them only within the study, and 8 only outside of the TelAbortion study. Most clinics generally followed a published protocol,^12^ with modifications determined by each individual clinic. The clinical protocols are described in the eTable in the Supplement. Most (n = 9) clinics offered no-test medication abortion up to 77 days of gestation. Some clinics (n = 10) varied the number of misoprostol tablets dispensed by pregnancy duration or study participation; others routinely provided 8 tablets to all patients (n = 4). More than half of clinics (n = 9) offered multiple methods of mifepristone dispensing during the study period; 3 dispensed mifepristone to patients only in-person, and 2 dispensed only by mail, either directly from the clinic or via mail-order pharmacy. Among the 12 clinics that dispensed mifepristone in person, 4 offered a curbside pickup option that allowed patients to receive the medications without coming inside the facility. Four clinics did not require Rh factor testing for any patients. All but 1 clinic routinely called, texted, or emailed patients at 1 to 2 weeks and again at 4 weeks to determine patient outcomes, while 1 clinic offered an optional follow-up contact or visit and advised patients to take a pregnancy test at 4 to 5 weeks to confirm completion.

### Description of the Sample

We received data on 4156 medication abortions. A total of 377 medical records were excluded because medical record data were invalid or incorrectly entered (n = 15), patients were treated outside the study period (n = 38), patients had screening ultrasonography (n = 279), patients did not take both mifepristone and misoprostol (n = 42), and patients were from the excluded clinic (n = 3). Some patients subsequently were entirely lost to follow-up and some provided incomplete follow-up data, for example, confirmation that they took the mifepristone and/or misoprostol but their medical records did not have sufficient data to determine whether their abortions were complete. Thus, of the remaining 3779 patients with abortions, 2825 (74.8%) had some follow-up data, and 2397 (63.4%) had abortion outcome data (Figure).

**Figure.**
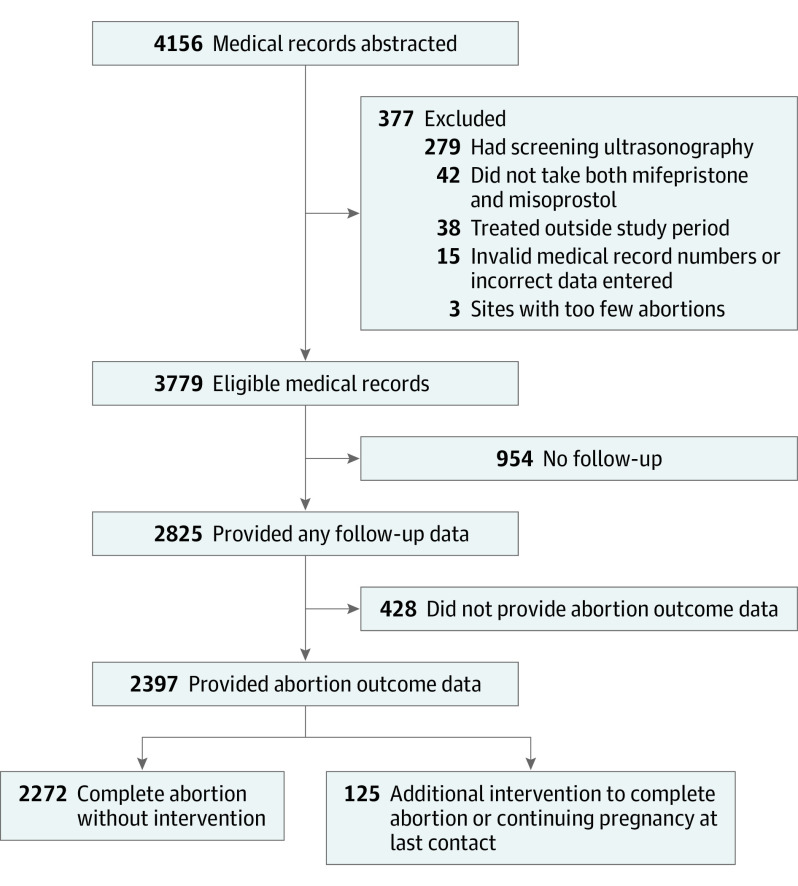
Study Flow Diagram

The sample was racially and ethnically diverse and included 870 (23.0%) Black patients, 533 (14.1%) Latinx/Hispanic patients, 1623 (42.9%) White patients, and 327 (8.7%) who identified as multiracial or with other racial or ethnic groups; race or ethnicity was unknown for 426 (11.3%) (Table 1). Half of the patients (1903 [50.4%]) paid for the abortion, at least in part, out of pocket. Patients lived in 34 states, and 2785 (73.7%) lived in urban areas. Nearly all abortions (3711 [98.2%]) were determined by patient history to be provided at 70 or less days of gestation. For 2511 (66.4%) abortions, the medications were dispensed in person, and for 1268 (33.6%), they were mailed to the patient. A total of 22 patients had more than 1 abortion at the same clinic and were represented more than once in the data set. Abortion outcomes were more likely to be known if the patient used private insurance to pay for the abortion, had lower pregnancy duration at treatment, received the abortion medications in person, or participated in the TelAbortion study. Among the 425 TelAbortion Study participants in this analysis, outcomes of 346 were reported in a previously published paper.^7^ Additionally, we included outcomes of 117 patients^14^ and 111 patients^8^ reported in previous articles.

**Table 1.** Characteristics of the Overall Sample and the Subgroup With Follow-up Data

Characteristic	No. (%)			*P* value
	Total eligible sample (n = 3779)	Sample with known abortion outcome (n = 2397)	Sample without known abortion outcome (n = 1382)	
Age at mifepristone provision, y				
≤24	1186 (31.4)	761 (31.7)	425 (30.8)	.23
25-34	1904 (50.4)	1206 (50.3)	698 (50.5)	
≥35	689 (18.2)	430 (17.9)	259 (18.7)	
Race and ethnicity				
Black	870 (23.0)	536 (22.4)	334 (24.2)	.008
Latinx/Hispanic	533 (14.1)	346 (14.4)	187 (13.5)	
White	1623 (42.9)	1034 (43.1)	589 (42.6)	
Multiracial or other^a^	327 (8.7)	223 (9.3)	104 (7.5)	
Unknown	426 (11.3)	258 (10.8)	168 (12.2)	
Residence^b^				
Urban	2785 (73.7)	1702 (71.0)	1083 (78.4)	.98
Suburban	243 (6.4)	153 (6.4)	90 (6.5)	
Rural	607 (16.1)	430 (17.9)	177 (12.8)	
Unknown	144 (3.8)	112 (4.7)	32 (2.3)	
Payment methods^c^				
Private insurance	769 (20.3)	546 (22.8)	223 (16.1)	<.001
Medicaid	881 (23.3)	581 (24.2)	300 (21.7)	.08
Abortion fund	212 (5.6)	140 (5.8)	72 (5.2)	.95
Patient	1903 (50.4)	1111 (46.3)	792 (57.3)	.10
Site subsidy	46 (1.2)	21 (0.9)	25 (1.8)	.13
Unknown	274 (7.3)	217 (9.1)	57 (4.1)	<.001
Previous medication abortion				
None	2626 (69.5)	1696 (70.8)	930 (67.3)	.002
Any	748 (19.8)	450 (18.8)	298 (21.6)	
Unknown	405 (10.7)	251 (10.5)	154 (11.1)	
Participated in the TelAbortion study				
No	3354 (88.8)	2031 (84.7)	1323 (95.7)	<.001
Yes	425 (11.2)	366 (15.3)	59 (4.3)	
Method of mifepristone provision				
In person	2511 (66.4)	1670 (69.7)	841 (60.9)	<.001
Mailed	1268 (33.6)	727 (30.3)	541 (39.1)	
No. of misoprostol tablets initially prescribed				
4 × 200 μg	2034 (53.8)	1267 (52.9)	767 (55.5)	<.001
8 × 200 μg	1745 (46.2)	1130 (47.1)	615 (44.5)	
Method of misoprostol provision				
Dispensed with mifepristone	3766 (99.7)	2386 (99.6)	1380 (99.9)	.85
Prescription	12 (0.3)	10 (0.4)	2 (0.1)	
Unknown	1 (0.0)	1 (0.0)	0 (0.0)	
Pregnancy duration by last menstrual period at mifepristone provision, d^d^				
<43	1400 (37.0)	904 (37.7)	496 (35.9)	<.001
43-56	1700 (45.0)	1080 (45.1)	620 (44.9)	
57-70	611 (16.2)	365 (15.2)	246 (17.8)	
>70	62 (1.6)	43 (1.8)	19 (1.4)	
Unknown	6 (0.2)	5 (0.2)	1 (0.1)	

^a^ The “other” category included Alaska Native, American Indian, Asian, Asian Indian, Filipino, Guyanian, Guamanian/Chamorro, Japanese, Middle Eastern, Native American, Native Hawaiian, Pacific Islander, and Turkish.

^b^ Estimated based on patient zip codes using Rural-Urban Commuting Area codes data.^27^

^c^ Multiple payment options may apply.

^d^ For the mailed group, pregnancy duration was calculated based on the date the medications were mailed.

### Effectiveness

The overall adjusted effectiveness rate was 94.8% (95% CI, 93.6%-95.9%) (Table 2) and was not significantly different whether the patients picked up their medications (95.4%; 95% CI, 94.1%-96.7%) or received them by mail (93.3%; 95% CI, 90.7%-95.9%). Effectiveness rates were highest among patients who were younger than 24 years and among those with pregnancies of 56 days of gestation or less. The sensitivity analysis excluding cases in which the abortion was determined complete by history alone resulted in an adjusted effectiveness rate of 93.0% (95% CI, 91.2%-94.8%). The sensitivity analysis that included only those with a gestation of 9 weeks or less who initially received 800 μg misoprostol found an adjusted effectiveness rate of 95.7% (95% CI, 94.3%-97.2%). Among the sample, 46 abortions (1.7%; 95% CI, 1.1%-2.2%) were determined by ultrasonography to be continuing viable pregnancies after the initial medications were dispensed (Table 3). Among those, 36 were treated with a procedure or additional medication to complete the abortion, 1 had a complete abortion with 1600 μg of misoprostol, and 9 had no further known intervention, so the final outcome was recorded as continuing viable pregnancy.

**Table 2.** Unadjusted and Adjusted Rate of Complete Medication Abortion by Characteristics of the Sample

Characteristic	Unadjusted complete case effectiveness rate, % (95% CI)	*P* value	Adjusted imputed effectiveness rate, % (95% CI)^a^	*P* value
No.	2397	NA	3779	NA
Overall	94.5 (92.9-96.1)	NA	94.8 (93.6-95.9)	NA
Age at mifepristone provision, y				
<24	96.4 (94.8-98.0)	[Reference]	96.6 (95.3-98.0)	[Reference]
25-34	94.1 (92.2-96.8)	.01	94.3 (92.8-95.9)	.02
≥35	92.8 (90.0-95.6)	.004	92.6 (89.9-95.3)	.002
Race and ethnicity				
Black	93.8 (91.2-96.5)	.88	93.9 (91.5-96.2)	.43
Latinx/Hispanic	96.4 (94.1-98.7)	.13	96.3 (94.1-98.6)	.31
White	94.0 (92.0-96.1)	[Reference]	94.9 (93.4-96.4)	[Reference]
Multiracial or other^b^	93.8 (90.1-97.5)	.90	93.7 (90.1-97.3)	.53
Residence				
Urban	94.0 (91.6-96.5)	[Reference]	94.2 (91.9-96.5)	[Reference]
Rural	94.7 (93.0-96.4)	.61	94.9 (93.6-96.2)	.57
Previous medication abortion				
None	94.6 (92.8-96.3)	[Reference]	94.7 (93.4-95.9)	[Reference]
Any	95.1 (92.6-97.5)	.69	95.1 (92.9-97.3)	.70
Participated in the TelAbortion study				
No	94.1 (92.1-96.0)	[Reference]	94.4 (93.2-95.8)	[Reference]
Yes	96.0 (93.9-98.2)	.14	96.3 (94.3-98.2)	.17
Method of mifepristone provision				
In person	94.9 (93.1-96.7)	[Reference]	95.4 (94.1-96.7)	[Reference]
Mailed	94.0 (91.8-96.3)	.54	93.3 (90.7-95.9)	.14
Pregnancy duration, d				
<43	95.2 (93.4-97.1)	[Reference]	95.5 (94.1-97.0)	[Reference]
43-56	95.2 (93.4-97.0)	.97	95.5 (94.1-96.9)	>.99
≥57	90.9 (87.1-94.6)	.004	90.9 (87.6-94.2)	.003

^a^ Multivariable estimates are adjusted for all other covariates in the table. Both models are mixed-effects models estimated with clinic as a random intercept.

^b^ The “other” category included Alaska Native, American Indian, Asian, Asian Indian, Filipino, Guyanian, Guamanian/Chamorro, Japanese, Middle Eastern, Native American, Native Hawaiian, Pacific Islander, and Turkish.

**Table 3.** Medication Abortion Additional Interventions and Major Adverse Events

Intervention/adverse event	No.	% (95% CI)	
		Unadjusted rate	Adjusted imputed rate^a^
**Effectiveness**			
No.	NA	2397	3779
Complete abortion without known intervention^b^	2272	94.5 (92.9-96.1)	94.8 (93.6-95.9)
Intervention to complete abortion^c^^,^^d^	116	5.1 (3.7-6.5)	4.6 (3.7-5.5)
Aspiration, second trimester abortion procedure, or surgery^e^	88	3.8 (2.8-4.8)	3.6 (2.7-4.4)
Prescribed >1600 μg of misoprostol, mifepristone, or other medications	37	1.7 (0.7-2.8)	2.5 (1.5-3.6)
Treatment for ectopic pregnancy	4	0.17 (0.00-0.33)	0.22 (0.00-0.45)
Continuing viable pregnancy without known intervention	9	0.34 (0.00-0.74)	1.27 (0.00-2.64)
**Safety**			
No.	NA	2825	3779
No major abortion-related adverse events	2813	99.6 (99.3-99.8)	99.5 (99.1-99.8)
Major abortion-related adverse events^2^	12	0.42 (0.18-0.66)	0.54 (0.18-0.90)
Blood transfusion	8	0.28 (0.02-0.53)	0.40 (0.08-0.73)
Other major surgery, including treatment of ectopic pregnancy	3	0.11 (0.00-0.23)	0.23 (0.00-0.51)
Hospital admission	6	0.21 (0.04-0.38)	0.21 (0.04-0.38)

Abbreviation: NA, not applicable.

^a^ Adjusted rates were calculated from mixed-effects logistic regression models fit to imputed data and adjusted for age, race and ethnicity, residence, prior medication abortion, participation in the TelAbortion Study, method of mifepristone provision, and pregnancy duration, with the exception of rare outcomes (n ≤ 9), which were not adjusted to facilitate model convergence.

^b^ Includes patients who received 800 μg of misoprostol initially followed by a single additional dose of 800 μg of misoprostol. One of these patients had a continuing viable pregnancy after the first dose and then aborted after the second.

^c^ Includes 36 continuing viable pregnancies after initial treatment. In total, there were 46 (1.7%; 95% CI, 1.1%-2.2%) continuing viable pregnancies after initial medications were dispensed.

^d^ Intervention and safety categories are not mutually exclusive because multiple interventions or treatments may have been provided for a single abortion.

^e^ Includes 2 dilation and evacuation procedures and 1 salpingectomy.

### Safety

The adjusted rate of major abortion-related adverse events was 0.54% (95% CI, 0.18%-0.90%) and was not statistically significantly different for patients who received medications in-person (0.46%; 95% CI, 0.09%-0.83%) and by mail (0.76%; 95% CI, 0.00%-1.57%). Among the patients with follow-up data, 12 major abortion-related adverse events occurred. These events included 8 blood transfusions, 3 major surgical procedures (including a possible but unconfirmed laparoscopic procedure for ectopic pregnancy), and 6 hospital admissions. Abortion-related hospital admissions included 1 due to pain that was treated with antibiotics and an unknown procedure to remove blood clots and residual tissue from the uterus; 2 for bleeding and pain that were treated with aspiration and transfusion; 1 for ongoing bleeding and kidney infection, which was treated with an unknown surgery, transfusion, and antibiotics; 1 for pain associated with endometritis that was treated with antibiotics; and 1 for major surgery (salpingectomy) to treat an ectopic pregnancy (Table 3). In total, 72 patients (2.6%; 95% CI, 1.9%-3.4%) were known to have visited emergency departments, including the 6 that resulted in hospital admissions described above.

We identified 4 ectopic pregnancies (0.22%; 95% CI, 0.00%-0.45%). One was detected 9 days after mifepristone ingestion after the patient contacted the clinic about unilateral pelvic cramping; the patient was admitted that day to a hospital where a salpingectomy was performed. Two were diagnosed after rising serum β-hCG results and treated with methotrexate; 1 of these may have had a laparoscopic procedure in addition, but full documentation was unavailable. The fourth was reported by an outside physician, but the treatment was unknown and documentation was unavailable.

During follow-up, 9 (0.40%; 95% CI, 0.00%-0.84%) patients were found to have had pregnancy durations greater than 70 days at mifepristone dispensing although not identified at screening (Table 4). Of these patients, 6 had procedures to complete the abortion at 88 to 101 days of gestation, 2 had complete abortions without additional intervention at 16 and 33 weeks, and 1, who received mifepristone at 87 days, had a continuing viable pregnancy at last follow-up contact 3 days after screening. The patient who was estimated to be at 33 weeks of gestation delivered a stillborn fetus at home, brought the fetus to the clinic, and required no further medical care.

**Table 4.** Patients Determined at Follow-up to Have Had Pregnancy Durations Greater Than 70 Days at Mifepristone Provision

Pregnancy duration documented at mifepristone provision, d	Corrected pregnancy duration at mifepristone provision based on pregnancy duration found at follow-up, d	Pregnancy duration at follow-up appointment, d	Abortion outcome
57	74	91	Procedure
53	80	88	Procedure
72	82	94	Procedure
60	85	92	Procedure
56	87	90	Lost to follow-up after ultrasonography
55	89	101	Procedure
70	90	93	Procedure
68	107	112	Complete abortion without intervention
42	230	231	Complete abortion without intervention

## Discussion

In this retrospective cohort study of patients who obtained medication abortions with screening by history alone from 14 clinics across the US using a range of protocols, we found high effectiveness and safety rates. As the largest (to our knowledge) US study of this approach to date, it offers a generalizable picture of what medication abortion may look like as more primary care and other clinicians adopt history-based screening protocols in-person or remotely. The effectiveness rate of 95% is comparable to studies of medication abortion models with screening ultrasonography that found effectiveness rates of 93% to 98%.^24,30,31^ The major adverse event rate of 0.5% (95% CI, 0.2%-0.9%) was slightly higher than previous studies finding rates of 0.2% to 0.3%^31,32^ but not significantly different and still rare from a clinical perspective. This study found similarly high effectiveness and safety rates comparing patients who received medications in-person vs by mail.

Before the COVID-19 pandemic, the FDA-mandated Risk Evaluation and Mitigation Strategy required mifepristone to be dispensed only in physicians’ offices, clinics, or hospitals. The FDA allowed mailing temporarily in mid-2020 and made this change permanent in December 2021.^33^ This study adds support for these actions, as we found that mifepristone can be dispensed safely either in person or by mail. Additionally, the mifepristone label could be revised to explicitly state that ultrasonography or clinical examination is not required if pregnancy duration can be reasonably estimated by history and if no symptoms or risk factors for ectopic pregnancy are present.

One of the major obstacles to expanded provision of medication abortion with history-based screening alone is clinician concern about the ability to identify an ectopic pregnancy. In this study, the ectopic pregnancy rate of 2 per 1000 suggests that the screening procedures used by the participating clinics will not triage all patients with ectopic risks to ultrasonography before the abortion. However, the potential benefits of expanded access, increased convenience, and earlier treatment conferred by removing testing requirements may outweigh potential risks of delayed identification of ectopic pregnancies.^34,35,36^

The risk of inadvertently treating a patient with a pregnancy duration greater than the accepted limit for medication abortion (70 or 77 days) is another concern. In this study, 9 patients were found at follow-up to have been treated at a gestation of greater than 70 days. The true number may be larger, as some advanced gestations may not have been recognized among patients who had successful abortions. We observed 1 case for which pregnancy duration was substantially underestimated; this patient passed the pregnancy and had a complete abortion at 33 weeks of gestation without additional intervention, medical complications, or sequelae. Clinicians offering the no-test approach should counsel patients about the possibility of an unidentified advanced gestation and discuss options for further evaluation and care if the patient were to encounter this situation.

### Limitations

The retrospective design of this study limited the precision and detail of the data. In particular, sites did not have uniform, validated criteria for confirming complete abortion using symptoms alone without ultrasonography, serum β-hCG, or urine pregnancy tests. However, the sensitivity analysis that excluded abortion outcomes determined without any tests found only slightly lower effectiveness rates than the full analysis. This study did not include a direct comparison group of patients who received pretreatment ultrasonography and other screening tests; however, given the similarity of our effectiveness and safety findings to those from multiple prior studies, we can be assured that there are not large differences in effectiveness and safety.

The follow-up rate of 75% was comparable to the 77% follow-up rates reported in previous research,^37,38^ but we may have failed to identify some additional interventions and adverse events. We aimed to mitigate the loss to follow-up with multiple imputation methods. If patients who were lost to follow-up were more likely to require additional intervention, our analysis may overestimate effectiveness. However, much of the loss to follow-up may be explained by the ubiquity of urine pregnancy tests that allow patients to assess their own outcomes. As history-based screening, telehealth, and self-assessment of outcomes using urine pregnancy tests become more common, the percentage of patients who do not experience complications and who contact the original clinician to confirm abortion completion is likely to decline. The net effect may be an apparent decline in effectiveness rates among patients who complete follow-up.

## Conclusions

Given the high effectiveness and very low risks associated with omitting in-person tests and using history-based screening alone, no-test medication abortion can offer substantial benefits to clinicians and patients and is consistent with the principle of patient-centered care. The use of history-based screening may appeal to primary care and other types of clinicians without access to ultrasonography technology or other tests. A shift toward history-based screening could expand the provision of abortion care to a variety of primary care clinicians, including nurse practitioners and physicians in family medicine, adolescent medicine, and internal medicine. Because many of these clinicians work in rural and low-income communities and with marginalized racial and ethnic groups and other historically marginalized communities, increasing the types of clinicians and locations offering abortion services could also lead to more equitable access to abortion care. This expansion could enhance patient-centered care and satisfaction, given that many patients would prefer to get their abortion from their primary care clinician.^39,40,41^

Combining history-based screening protocols with a pharmacy prescription or mailing of abortion medications to patients can support public health efforts and reduce the numerous logistical, distance, and cost barriers to abortion.^21,22,42,43^ No-test medication abortion can lower costs, result in earlier treatment, increase convenience and privacy, and allow patients to avoid harassment at clinics.^17,35^ These benefits have the potential to increase equitable access to abortion for all patients, particularly marginalized racial and ethnic groups, those with low incomes, who reside in rural locations, and who face greater barriers to abortion care. Clinicians should eliminate unnecessary tests to support patient-centered care.

## Notes

Supplement. **eTable.** Service delivery characteristics of the participating clinics and study population
