# Supplementary material for: Outcomes and Safety of History-Based Screening for Medication Abortion: A Retrospective Multicenter Cohort Study
Source: JAMA Intern Med. 2022 Mar 21;182(5):482–91. doi: 10.1001/jamainternmed.2022.0217 (PMC8938895; doi:10.1001/jamainternmed.2022.0217)
Supplement: Supplement. — eTable. Service delivery characteristics of the participating clinics and study population [file jamainternmed-e220217-s001.pdf]

Supplemental Online Content

Upadhyay UD, Raymond EG, Koenig LR, et al. Outcomes and safety of history-based screening for medication abortion: a retrospective multicenter cohort study. *JAMA Intern Med*. Published online March 21, 2022. doi:10.1001/jamainternmed.2022.0217

eTable. Service delivery characteristics of the participating clinics and study population

This supplemental material has been provided by the authors to give readers additional information about their work.

© 2022 Upadhyay UD et al. *JAMA Internal Medicine.*

eTable. Service delivery characteristics of the participating clinics and study population	
	N=14
**Clinic Characteristics**	
Clinic Type	
Independent abortion clinic	4
Planned Parenthood affiliate	4
University-affiliated or academic clinic	4
Online only service	2
Participated in the TelAbortion Study	
No	8
Yes	6
**Eligibility**	
Criteria used to determine eligibility for no-test medication abortion	
Patient certain of last menstrual period date	14
No recent pelvic pain	12
No recent vaginal bleeding	13
No previous ectopic pregnancy	12
No previous sterilization	10
No IUD in place at or since conception	14
No previous pelvic inflammatory disease	8
No previous c-section	0
Pregnancy duration limit for no-test medication abortion	
70 days	5
77 days	9
Clinic policy for offering no-test medication abortion to patients	
All eligible patients	12
By provider discretion	2
Routine pre-abortion tests conducted for no-test medication abortion patients	
Abdominal Exam	0
Serum β-hCG	0
Hemoglobin or hematocrit	0
COVID-19	0
**Rh Immune Globulin**	
Does not administer RhIg to any Rh- patients	4
RhIg recommended/required for Rh- patients over a certain pregnancy duration	10
**Mifepristone and Misoprostol Dispensing**	
Method of mifepristone dispensing	
Only in-person	3
All pills mailed	2
Offered both in-person and mailing	9
Curbside pick-up available (among clinics that offered in-person dispensing)	
No	8
Yes	4
Method of misoprostol dispensing	
Dispensed with mifepristone	12
Prescription filled at pharmacy	1

© 2022 Upadhyay UD et al. *JAMA Internal Medicine.*

Varied	1
Number of misoprostol tablets routinely provided	
8 x 200 *μg*	4
Varied by pregnancy duration	10
**Follow-up**	
Earliest scheduled patient interaction in routine follow-up plan	
No scheduled follow-up visit	1
<7 days	11
1-2 weeks	2

hCG – human chorionic gonadotropin
Rh – Rhesus
RhIg – Rhesus Immune Globulin
μg – micrograms

© 2022 Upadhyay UD et al. *JAMA Internal Medicine.*
